# Resting-State Electroencephalogram (EEG) as a Biomarker of Learning Disabilities in Children—A Systematic Review

**DOI:** 10.3390/jcm14165902

**Published:** 2025-08-21

**Authors:** James Chmiel, Jarosław Nadobnik, Szymon Smerdel, Mirela Niedzielska

**Affiliations:** 1 Faculty of Physical Culture and Health, Institute of Physical Culture Sciences, University of Szczecin, Al. Piastów 40B Blok 6, 71-065 Szczecin, Poland; 2 School of Sports Championschips, FASE—Football Academy School of Excellence, 71-241 Szczecin, Poland; 3 Department of the History of Medicine and Medical Ethics, Pomeranian Medical University, 70-204 Szczecin, Poland

**Keywords:** learning disability, learning disabilities, learning difficulties, learning problems, EEG, electroencephalography, electroencephalogram, brain oscillations, QEEG, neurophysiology, neural correlates, maturational delay

## Abstract

**Introduction:** Learning disabilities (LD) compromise academic achievement in approximately 5–10% of school-aged children, yet the neurophysiological signatures that could facilitate earlier detection or stratification remain poorly defined. Resting-state electroencephalography (rs-EEG) offers millisecond resolution and is cost-effective, but its findings have never been synthesized systematically across pediatric LD cohorts. **Methods:** Following a PROSPERO-registered protocol (CRD420251087821) and adhering to PRISMA 2020 guidelines, we searched PubMed, Embase, Web of Science, Scopus, and PsycINFO through 31 March 2025 for peer-reviewed studies that recorded eyes-open or eyes-closed rs-EEG using ≥ 4 scalp electrodes in children (≤18 years) formally diagnosed with LD, and compared the results with typically developing peers or normative databases. Four reviewers independently screened titles and abstracts, extracted data, and assessed the risk of bias using ROBINS-I. **Results:** Seventeen studies (704 children with LD; 620 controls) met the inclusion criteria. The overall risk of bias was moderate, primarily due to small clinic-based samples and inconsistent control for confounding variables. Three consistent electrophysiological patterns emerged: (i) a 20–60% increase in delta/theta power over mesial-frontal, fronto-central and left peri-Sylvian cortices, resulting in markedly elevated θ/α and θ/β ratios; (ii) blunting or anterior displacement of the posterior alpha rhythm, particularly in language-critical temporo-parietal regions; and (iii) developmentally immature connectivity, characterized by widespread slow-band hypercoherence alongside hypo-connected upper-alpha networks linking left-hemisphere language hubs to posterior sensory areas. These abnormalities were correlated with reading, writing, and IQ scores and, in two longitudinal cohorts, they partially normalized in parallel with academic improvement. Furthermore, a link between reduced posterior/overall alpha and neuroinflammation has been found. **Conclusions:** Rs-EEG reveals a robust yet heterogeneous electrophysiological profile of pediatric LD, supporting a hybrid model that combines maturational delay with persistent circuit-level atypicalities in some children. While current evidence suggests that rs-EEG features show promise as potential biomarkers for LD detection and subtyping, these findings remain preliminary. Definitive clinical translation will require multi-site, dense-array longitudinal studies employing harmonized pipelines, integration with MRI and genetics, and the inclusion of EEG metrics in intervention trials.

## 1. Introduction

Learning disabilities (LD)—referred to as specific learning disorders in the DSM-5 and developmental learning disorders in the ICD-11—constitute a group of neurodevelopmental conditions that impair the efficient acquisition and use of reading, spelling, written expression, mathematics, or, less frequently, oral language [1,2]. They are considered specific because the difficulties are circumscribed rather than global and unexpected because they occur in children with average or above-average intelligence, intact vision and hearing, sufficient cultural exposure, and access to regular classroom instruction [3]. LDs are not transient delays; skill deficits persist for at least 6 months despite targeted support and significantly hinder academic progress, daily functioning, or future employment prospects [4]. Under the U.S. federal education law (IDEA) and most international policies, children qualify for services when low academic achievement is accompanied by (a) clinical judgment that the pattern is best explained by LDs and (b) exclusion of major sensory, neurological, or emotional disorders. LDs are divided into two reading domains: dyslexia, which occurs at the word level, and reading comprehension, which occurs at the text level. Math issues, which are frequently word problems, might be computational (dyscalculia) or include problem solving. Basic transcribing skills may be involved in difficulties with written expressions [5]. Another group of LDs are specific learning disorders (SLDs). DSM-5 defines them as “Difficulties learning and using academic skills indicated by the presence of one of the following that have persisted for at least 6 months, despite the provision of interventions that target those difficulties: slow/inaccurate/effortful reading, difficulty understanding what has been read, difficulty in spelling, difficulty in written expression, difficulty in mastering number sense, number facts, calculations, and difficulty with math reasoning” [6]. Another division of LDs is the division into verbal and nonverbal learning disabilities (VLDs and NVLD)s. There is great heterogeneity in the criteria used to define NVLDs. A deficit in visuospatial ability/intelligence was the most common criterion used, followed by discrepancy between verbal and nonverbal intelligence (VIQ>PIQ split of 10 or greater) [7].

Large population-based studies across North America, Europe, and Asia converge on point prevalence of 5–10% for any form of LD, making it the most common disability category among school-aged children [8]. Dyslexia alone affects 5–17.5%, while dyscalculia is reported in approximately 5% [8]. Males are diagnosed approximately twice as often as females, although this disparity narrows when referral bias is accounted for [9]. Symptom presentation evolves with age: preschoolers may exhibit delayed speech, poor phonological awareness, or difficulty grasping number concepts; children in early elementary grades often struggle with decoding, spelling, or basic arithmetic facts; adolescents may read slowly, produce disorganized written work, or fail higher-level mathematics [10]. Longitudinal and review studies indicate that these academic deficits rarely resolve spontaneously; without evidence-based intervention, LD is associated with reduced secondary school completion, lower tertiary education enrollment, and underemployment in adulthood—even after controlling for IQ and socioeconomic status [11,12].

Up to 50% of students with LD also meet diagnostic criteria for attention deficit/hyperactivity disorder (ADHD) [13], and 70% experience anxiety [14] or depressive symptoms (36%) [15] secondary to chronic academic failure. The social repercussions are cumulative: children may withdraw, act out, or adopt “learned helplessness.” Adolescents with untreated LD are at increased risk for school dropout [1], substance use [16], and involvement in the juvenile justice system [17]. These cascading effects support a public health perspective, emphasizing that early detection and comprehensive support not only improve academic outcomes but also enhance mental health and civic engagement.

Twin and family studies demonstrate substantial heritability, with estimates ranging from 0.40 to 0.70 for reading and math disabilities [18]. Molecular genetic studies have implicated several susceptibility genes in dyslexia (e.g., DCDC2, KIAA0319, DYX1C1) [19,20], many of which are involved in neuronal migration and axonal guidance during prenatal cortical development. Functional MRI (fMRI) studies show atypical activation patterns in the left-hemisphere reading network—including occipito-temporal, temporo-parietal, and inferior frontal gyri—in individuals with dyslexia [21,22], and disrupted intraparietal sulcus–prefrontal circuits in dyscalculia [23,24]. These neural anomalies typically emerge early, are detectable before formal schooling, and interact with environmental factors—such as language exposure, instructional quality, and socioeconomic adversity—to influence the severity of learning outcomes. Contrary to widespread myth, LD is not caused by poor vision, laziness, emotional trauma, or “left- versus right-brain” dominance.

Meta-analyses and systematic reviews confirm that intensive, explicit instruction—phonics-based programs for reading [25], systematic fact retrieval and strategy training for mathematics [26], and structured curricula for writing [27]—yield the most significant improvements. Effective interventions are characterized by daily small-group sessions, guided practice with immediate feedback, and continuous progress monitoring [28]. When implemented with fidelity, response-to-intervention (RtI) frameworks not only raise overall achievement but also reduce inappropriate referrals to special education. The results from the most recent meta-analysis reveal that phonics instruction is the most intensively investigated treatment approach. In addition, it is the only approach whose effectiveness on reading and spelling performance in children and adolescents with reading disabilities is statistically confirmed [29]. Given the lifelong persistence of LD, transitional planning, vocational accommodations, and adult literacy or numeracy programs remain critical components of long-term support.

LD is a neurodevelopmental condition that must be understood in order to inform effective treatment strategies. Like all developmental disorders, LD is characterized by atypical brain function. Modern neuroscience offers a range of neuroimaging methods that allow for the observation of brain activity and structure with high temporal or spatial resolution. As noted above, fMRI provides excellent spatial resolution, enabling the detailed mapping of brain structures and functions. However, its use is constrained by high cost, reduced temporal resolution, and limited availability.

The oldest and most fundamental neuroimaging technique is electroencephalography (EEG). EEG records millisecond-scale fluctuations in post-synaptic potentials at the scalp, giving it the highest temporal resolution of all non-invasive human neuro-imaging modalities used in humans [30]. When recordings are obtained while participants sit quietly with eyes open or closed, the signal is referred to as resting-state EEG (rs-EEG). Despite the absence of an explicit task, rs-EEG exhibits a rich structure, reflecting the brain’s intrinsic oscillatory and network dynamics [31].

The classical approach to analyzing rs-EEG involves decomposing the signal into canonical frequency bands (δ, θ, α, β, γ) and computing power spectral density (PSD). Band-limited power captures developmental changes and individual traits, and large normative databases—such as the Cuban Normative Dataset—now provide age- and sex-referenced z-scores [32]. In addition to local power, functional connectivity metrics—such as coherence, phase-lag index, imaginary coherence—are used to assess long-range interactions and are increasingly computed in source space to improve spatial specificity [31].

A complementary method segments rs-EEG into brief (80–120 ms) epochs of quasi-stable scalp topographies known as microstates. Four canonical classes (A–D) consistently emerge across individuals and species; their temporal parameters (mean duration, coverage, transition probabilities) have been linked to the activity of fMRI-defined resting-state networks and to cognitive function and psychopathology [33,34].

Because rs-EEG can be acquired quickly and at a low cost, there is growing interest in its potential for diagnostic or prognostic biomarker development. Recent studies have shown that PSD features, when combined with support-vector or Gaussian process classifiers, can distinguish first-episode psychosis from healthy controls with over 90% specificity [35]. Other systematic reviews have reported characteristic θ-↑/α-↓ shifts in conditions like chronic pain and pathological fatigue [36,37].

The aim of this systematic review is to identify and analyze studies that used EEG to measure resting-state EEG in children up to 18 years of age diagnosed with learning disabilities. Studies involving participants with comorbid disorders such as ADHD, dyslexia, and dyscalculia were excluded. We sought to identify consistent patterns of oscillatory activity in LD and to explore correlations between EEG findings and other variables (e.g., age, IQ). Additionally, we assess the methodologies used in the included studies and discuss their limitations. Finally, we outline future research directions and propose key questions for subsequent resting-state EEG in the context of learning disabilities.

## 2. Materials and Methods

**Figure 1:**
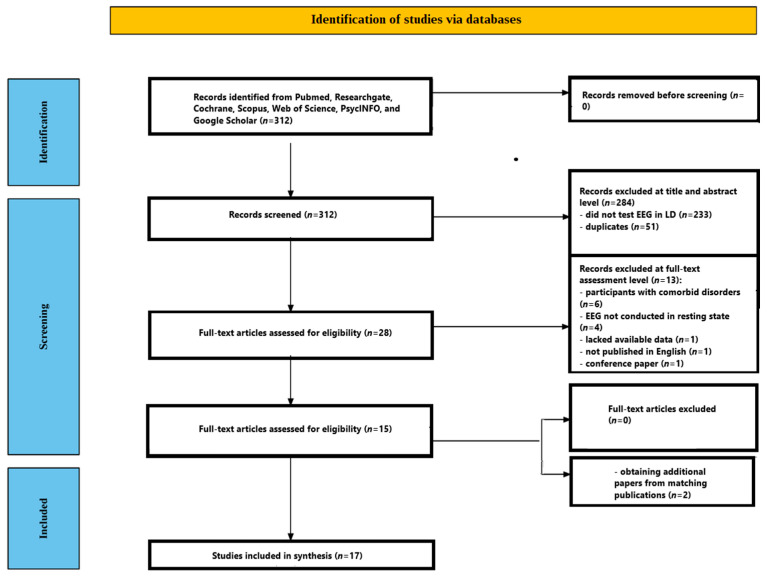
Flowchart depicting the different phases of the systematic review.

This systematic review was conducted and is reported in accordance with the PRISMA 2020 statement [38]. The study selection process is illustrated in the PRISMA flow diagram (Figure 1).

### 2.1. Protocol and Registration

The review protocol was drafted a priori, peer-reviewed by all authors, and prospectively registered with PROSPERO (registration No. CRD420251087821) on July 2025 [39]. No methodological deviations from the registered protocol occurred.

### 2.2. Eligibility Criteria and Study Selection

Eligibility criteria were defined a priori based on the PICOS framework.

(a) Population: We included empirical studies involving human participants from birth to 18 years of age who had a formal diagnosis of a learning disability—referred to as specific learning disorder in DSM-5 or developmental learning disorder in ICD-11 or ICD-10—established through clinical or psychoeducational assessment. To minimize confounding, we excluded samples in which participants presented with comorbid neurodevelopmental or neurological conditions such as ADHD, autism, epilepsy, intellectual disability, or acquired brain injury. No restrictions were placed on sex, ethnicity, or recruitment setting.
(b) Index (Exposure): Studies were required to acquire resting-state EEG (eyes-open, eyes-closed, or alternating) using a conventional clinical or research montage with at least 5 scalp electrodes. Investigations that relied exclusively on task-evoked/event-related, sleep, or ambulatory EEG, or that derived EEG indirectly from magnetoencephalography, were excluded.
(c) Comparators: Eligible studies compared children with learning disabilities to typically developing peers and/or included within-group correlational or longitudinal analyses. While a control group was desirable, it was not mandatory for inclusion.
(d) Outcomes: The review focused on resting-state quantitative EEG measures, including—but not limited to—band-limited power, peak frequency, functional connectivity indices (e.g., coherence, phase-lag index), and microstate parameters. Studies reporting only qualitative impressions or lacking extractable numerical data were excluded.
(e) Study Design and Publication Type: We included peer-reviewed original research employing cross-sectional, longitudinal, or quasi-experimental designs. Reviews, meta-analyses, editorials, conference abstracts without full manuscripts, single-case reports (fewer than five participants), and all animal or in vitro studies were excluded.
(f) Language, Period, and Availability: Only full-text articles published in English between 1 January 1980, and 31 April 2025, were considered. Non-English articles were excluded unless a complete, authoritative English translation was available.

These criteria were intentionally broad to capture the full range of contemporary rs-EEG research in pediatric learning disabilities while maintaining internal validity in accordance with PRISMA guidelines [38].

### 2.3. Information Sources and Search Strategy

Six bibliographic databases were searched from inception to 28 May 2025: PubMed/MEDLINE, Embase, Web of Science Core Collection, Scopus, PsycINFO, Google Scholar, and Researchgate. The search strategy combined controlled vocabulary and free-text terms: “learning disabilities” OR “learning disability” OR “learning problems” OR “learning difficulty” OR “learning difficulties” AND “electroencephalography” OR “electroencephalogram OR “EEG” or “QEEG”. Similar and citing studies suggested by PubMed algorithms were reviewed, and reference lists of included articles were hand-searched for additional relevant studies.

### 2.4. Selection Process

All retrieved records were imported into EndNote™ X9 and de-duplicated automatically and manually. Three reviewers (J.C., J.N. and S.S) independently screened titles and abstracts, followed by full-text assessment against the eligibility criteria. Discrepancies were resolved through consensus or, if necessary, adjudicated by a fourth reviewer (M.N.).

### 2.5. Data Extraction Procedure

A standardized extraction form (piloted on three studies) was used to capture information on study design, sample size, age and IQ, LD subtype, EEG acquisition parameters (montage, reference, sampling rate, state), quantitative EEG metrics, and main findings. Two reviewers extracted data independently; discrepancies were resolved through discussion. Corresponding authors were contacted twice to request missing numerical data.

### 2.6. Risk-of-Bias Assessment

Because all included studies were observational, risk of bias was assessed using the ROBINS-I tool [40]. Each domain was rated as low, moderate, or high risk; the overall rating was determined by the highest domain rating. Risk-of-bias assessments were conducted independently by two reviewers, with disagreements resolved through arbitration as described above.

## 3. Results

### 3.1. Study Selection

Following deduplication, 312 records were identified. Based on title and abstract screening, 28 full-text articles were reviewed. Of these, 6 studies were excluded due to the inclusion of participants with comorbid disorders (e.g., ADHD, dyslexia, intellectual disability), 4 were excluded because EEG was not recorded in a resting-state condition, 1 lacked available data, 1 was published in a language other than English, and 1 was a conference paper. After full-text screening, 15 studies met the inclusion criteria. An additional 2 studies were identified through citation tracking of included articles, resulting in a total of 17 studies being included in the final review [41,42,43,44,45,46,47,48,49,50,51,52,53,54,55,56,57].

The studies included are presented in Table 1. While varying in scope and design, all examined resting-state EEG or QEEG features in children with LD. Below, we organize their collective findings according to (a) study designs, (b) participant samples, (c) methodological approaches, (d) data analysis techniques, (e) key EEG abnormalities observed, and (f) relationships with academic performance and IQ.

### 3.2. Study Designs

Most studies employed cross-sectional designs to compare children with LD—or closely related clinical classifications—to typically developing peers at a single time point (e.g., [42,44,46,52]). These studies generally aimed to identify distinct neurophysiological profiles, with a common emphasis on elevated slow-wave activity (delta, theta) in LD samples. A smaller subset of studies adopted longitudinal designs, tracking participants across multiple years [49,50,55]. This approach allowed researchers to determine whether EEG markers shift toward more typical patterns over time (i.e., “maturational lag”) or remain consistently atypical. Longitudinal findings indicated that while some children with LD showed partial normalization of slow-wave features, others retained significant EEG deviations across development.

### 3.3. Participant Samples

All samples comprised school-aged children, primarily between the ages of 6 and 16, with minor variations in mean age (e.g., ~9–12 in [41], 7–12 in [47], up to 16 in [43]). Participants were recruited from elementary or middle schools, specialized educational programs, or clinical settings (e.g., neurology clinics).

Definitions of “learning disability” varied across studies. Some relied on formal diagnoses based on DSM criteria [44,52], while others used educational placement criteria [42,47] or combined IQ thresholds with documented academic underachievement [41,52]. Certain studies differentiated between LD subtypes, such as “LD-NOS” vs. “LD-Verbal” [41] or “SLD” vs. “generalized LD” [45], whereas others grouped participants based on reading and writing categories [49,50,51]. This variability highlights the inherent heterogeneity of LD research.

Most studies excluded children with overt neurological or psychiatric conditions such as epilepsy or ADHD [41,48,56]. However, a few acknowledged the presence of mild attentional difficulties within LD samples [45,52], reflecting the real-world overlap between LD and attention deficits. IQ cutoffs were commonly applied to exclude intellectual disability, typically requiring scores above 70–85. One multi-center home-acquisition cohort enrolled 100 children with LD (Mage = 8.75; 80M/20F) and 100 TD controls (Mage = 8.85; 80M/20F), all Caucasian, diagnosed by psychiatrists using DSM-5, medication-free and without ADHD/autism [57].

### 3.4. Methodological Approaches

All included studies employed resting-state EEG protocols, with most recordings under eyes-closed (EC) conditions; some studies also included eyes-open (EO) sessions [41,56]. The number of electrode channels ranged from as few as 6 [47] to the full 19-channel 10–20 montage or denser arrays [41,52,56]. Recording durations varied, but most protocols collected at least 20–60 s of artifact-free data per child. Standard preprocessing steps included the removal of ocular, muscular, and movement artifacts, typically via visual inspection or independent component analysis (ICA).

Spectral analysis was the predominant method, most commonly employing Fast Fourier Transform (FFT) to compute power within canonical frequency bands (delta, theta, alpha, beta). Some studies further subdivided these bands (e.g., alpha1 vs. alpha2, or beta1 vs. Beta2) [46,53]. Several studies also calculated ratio metrics (e.g., theta/beta, theta/alpha) to characterize developmental or attentional profiles [41,45]. Advanced source localization techniques (e.g., sLORETA, FD-VARETA) were used in a few studies to estimate intracortical generators of EEG signals [44,56]. Others examined functional connectivity using coherence or phase synchrony metrics [49,50,51], while a subset applied multivariate neurometric methods to assess whether EEG profiles could distinguish LD from typical development with diagnostic precision [39,48]. Beyond laboratory 10–20 systems, one study used a consumer 14-channel EMOTIV EPOC-X headset in a two-minute eyes-open resting-state protocol collected at home, with ~50 sessions per participant (10,041 sessions total). Signals were filtered (0.5–45 Hz; 50 Hz notch) and downsampled to 128 Hz; delta was not recorded. Z-scores were computed manually. The device lacks ICA-based artifact rejection, and the authors note potential EMG contamination in beta/gamma bands. That study also implemented supervised machine learning (ANN/MLP) on 70 band-power features, reporting AUC ≈1.0 and accuracy ≈98.5–99.5% with k-fold cross-validation and an independent test set, and provided channel-by-channel correlation matrices. Given repeated sessions per participant, subject-wise data partitioning is essential to avoid information leakage; the authors describe both cross-validation and a held-out test set, though session-vs. participant-level folds are not fully detailed [57].

### 3.5. Data Analysis and Interpretation

A common analytic strategy involved adjusting EEG power for age, as slow-wave activity (delta, theta) typically diminishes during neurodevelopment. Some studies generated age-based z-scores or applied developmental regression equations to identify individuals whose power levels deviated significantly from expected norms [42,43].

Several investigations conducted subgroup analyses within their LD samples. Cluster analysis and discriminant function approaches revealed distinct cognitive or neurophysiological subtypes. These often showed that while some children with LD exhibited pronounced deficits in language-associated brain regions, others displayed broader, more diffuse abnormalities [45,52,53]. Direct correlations between EEG power—particularly in alpha and theta bands—and cognitive measures (e.g., IQ, reading, and math performance) were also reported. These findings suggest that neurophysiological immaturity or hyperconnectivity in certain frequency bands may reliably predict poorer academic achievement [46,52].

Longitudinal studies linked age-related EEG patterns to the developmental trajectories of learning difficulties. Although some children showed EEG shifts toward typical maturation—such reduced slow-wave power or strengthened alpha networks—others maintained atypical profiles, suggesting persistent delay or divergent neurodevelopmental pathways [49,50,55]. Overall, the findings consistently demonstrate that resting-state EEG abnormalities, especially in theta and alpha power or connectivity, are strongly associated with LD status and can help parse subtypes of learning challenges based on underlying neurophysiological mechanisms.

Consistent with prior work using discriminant/neurometric models, the machine-learning approach in Eroğlu (2025) achieved near-ceiling classification of LD vs. TD using resting-state band powers (theta/alpha/gamma). While these results underscore the diagnostic potential of rs-EEG feature sets, interpretability is tempered by methodological constraints (EO-only data, no delta, consumer hardware, limited artifact control, and possible session-level cross-validation effects) [57].

### 3.6. Key Observed EEG Abnormalities

Across the sixteen resting-state investigations, a highly consistent electrophysiological phenotype emerges for children with LD, despite variability in sample characteristics, EEG methodology, and analytic approaches.

#### 3.6.1. Pervasive Excess of Slow-Wave Activity (δ, θ) and Elevated Spectral Ratios

All power-spectral studies reported a reproducible excess of slow oscillations in children with LD. Absolute theta power was typically 30–60% higher than in age-matched controls, with delta power elevated by approximately 20–40% [41,42,46,47,52,56]. When adjusted for age and expressed as z-scores, the mean shift ranged from +0.5 to +1.5 SD, equivalent to an electrodevelopmental delay of two to four years in children aged 6 to 12—based on neurometric growth curves and international norms [43,50,55].

This amplification of slow-wave activity drives the classic maturational indices sharply upward. Theta/alpha and theta/beta ratios (TAR, TBR) at frontal and midline leads often exceeded 3.5 in LD cohorts—levels rarely observed in neurotypical children over eight years of age—and distinguished LD from controls with 70–85% sensitivity and specificity in clinical samples [41,45,46]. In Jancke’s independent component analysis, the mean frontal TAR reached 3.9 (controls = 2.2) and TBR reached 4.2 (controls = 2.6), representing increases of 60–80% across frontal, central, and parietal cortices [41]. A subsequent replication using whole-head phase analysis reported large effect sizes (Cohen’s d ≈ 0.8–1.1) for theta excess at nearly every electrode [56].

Topographically, the excess follows a consistent distribution. Fronto-central maxima predominate, reflecting immaturity in executive and cingulo-cortical circuits; left fronto-temporal peaks are more prominent in verbally defined LD subtypes, while parieto-occipital foci are observed in children with more generalized scholastic impairments [41,43,54]. These spatial patterns were evident regardless of reference montage or analytic method. Converging evidence from FFT, group-ICA, FD-VARETA source imaging, and developmental z-mapping consistently identified slow-wave generators in the mesial frontal cortex, superior temporal gyrus, and posterior parietal lobule [41,44,56].

The magnitude of the slow-wave surplus increased under cognitive load. During challenging visuospatial tasks, right fronto-temporal theta rose by an additional 15 percentage points in boys with LD—suggesting not global hypo-arousal but rather cortical resource overload [47]. Conversely, task-induced alpha blocking was attenuated, and slow-to-fast spectral ratios remained pathologically elevated, implying that cortical systems failed to accelerate rhythmically in response to processing demands.

Clinically, every 1-SD elevation in theta power was associated with an estimated drop of two reading grade equivalents. Children whose theta exceeded +1.5 SD at baseline consistently remained below academic benchmarks three years later, even in the context of general maturational improvement [55]. Thus, the fronto-centro-parietal δ/θ surplus—and derived TAR/TBR indices—constitutes a robust electrophysiological fingerprint of LD at rest, likely reflecting a combination of delayed cortical pruning, inefficient top-down regulation, and region-specific network dysmaturation.

Note that one EO, consumer-device study did not record delta and therefore cannot inform δ-band effects [57].

#### 3.6.2. Attenuation or Topographic Reversal of Posterior α Power

In typically developing children, resting-state eyes-closed EEG shows a *robust* posterior dominant rhythm: upper-alpha activity (≈10–12 Hz) peaks over the parieto-occipital cortex and decreases sharply toward the frontal regions. At least eight of the reviewed studies report that this posterior gradient is blunted—or even reversed—in children with learning disabilities.

Source-level tomography offers the clearest insight into this phenomenon. Using FD-VARETA, Fernández et al. found that, across the 9.75–12.87 Hz range, occipital current density in controls exceeded that of children with LD-NOS by approximately 30%, with maximal group separation observed in the right lingual and cuneus gyri; notably, no LD child exhibited a typical occipital “alpha hotspot” [44]. A parallel sLORETA decomposition in Jäncke’s gICA dataset revealed that the component anchored in primary and secondary visual cortices (gIC-3) contributed significantly *less* alpha power in the LD group than in controls, while frontal midline sources remained unaffected—confirming a selective posterior deficit [41].

Scalp-level analyses converge on this topographic pattern. In a Brazilian clinic cohort, Fonseca et al. reported a 15–25% reduction in relative α_2_ power (10.1–12.5 Hz) at parietal and occipital electrodes (*p* < 0.0033 after Bonferroni correction), accompanied by a 7–10% increase at F3/F4, effectively flattening the typical back-to-front gradient [46]. Similarly, a German epidemiological study found that α_2_ and β_2_ were attenuated at O1/O2 yet slightly elevated fronto-centrally, resulting in a significant Group × Region interaction (F ≈ 12, *p* < 0.0001) [42]. A large-scale discriminant analysis by Thatcher et al. confirmed this reversal: in 74% with LD, frontal amplitude exceeded parietal (F4 > P4), a pattern seen in only 18% of controls and strongly predictive of lower WRAT-Reading scores [52].

Functionally, diminished posterior alpha appears to index inefficiencies in sensory–perceptual systems that support language processing. In Harmony’s literacy study, children in the “very poor” reading group showed a 0.9 SD drop in alpha power over the left anterior–temporal electrodes (F7, T3) compared to good readers; this alpha deficit alone accounted for 48% of the variance in dictation and spontaneous writing scores, after adjusting for IQ and socioeconomic status (SES) [54]. Longitudinally, as children with mild learning difficulties improved academically, their parietal alpha activity approached typical levels. In contrast, those with severe impairments continued to exhibit low posterior alpha despite gains in faster rhythms, suggesting the persistence of a neural bottleneck [55].

The study [57] framed reduced posterior/overall alpha (within a developmentally shifted band) as part of a putative neuroinflammatory EEG signature, aligning conceptually with our observed blunting of the posterior alpha gradient.

Not all studies found generalized global alpha suppression. One large dataset from 2019 observed a modest increase in lower alpha (8–10 Hz) activity posteriorly in children with LD, but no corresponding change in the task-relevant upper-alpha band—emphasizing the need to separate sub-bands when interpreting results [56]. Nonetheless, the broader evidence indicates that children with learning disabilities frequently lose the typical occipital alpha dominance and, in many cases, exhibit an anterior shift in the alpha peak. This “topographic flip” complements the frontal slow-wave excess described earlier, revealing an electrophysiological pattern in which the cortex appears simultaneously over-slow in the front and under-idling in the back. This signature reflects immature visuo-temporal processing and disrupted posterior–frontal communication that underlie deficits in reading, writing, and related scholastic skills.

#### 3.6.3. Aberrant β Activity Reflecting Cortical Disinhibition or Compensatory Effort

Although findings in the β-band are less uniform than those in the slow-wave range, several converging patterns emerge when sub-bands and scalp topography are taken into account.

In the largest resting-state dataset to date, children with LD showed a marked frontal β surge: both lower-β (13–20 Hz) and upper-β (20.5–30 Hz) power at Fz and F4 were approximately 40–50% higher than in controls (d ≈ 0.7–0.9, *p* < 0.01) during eyes-open recordings, with no significant differences observed at posterior electrodes [56]. A similar but smaller anterior rise (≈10%) was reported in a German epidemiological study, where β_2_ (17.5–25 Hz) was slightly elevated fronto-centrally but *reduced* occipitally, yielding a significant Group × Region interaction (F ≈ 12, *p* < 0.0001) [42]. Source localization attributed this frontal excess to mesial dorsal frontal and anterior cingulate generators—regions that, when overactive at fast frequencies, are often interpreted as reflecting cortical disinhibition and heightened tonic effort to sustain executive control in the context of inefficient or dysregulated networks.

Under cognitive load, these β abnormalities become more pronounced. In Lubar’s seven-condition paradigm, boys with LD generated 10–15% more 20–28 Hz power than controls in 86% of the 672 site × task comparisons, with the most significant spikes over right fronto-temporal sites during challenging visuospatial puzzles [47]. Similarly, in Harmony’s longitudinal cohort, children with the most severe reading impairments exhibited elevated frontal β absolute power at baseline. These levels declined as their performance improved, implying that beta activity may reflect compensatory effort rather than a static neurophysiological trait [55].

Not all β anomalies involve increased power. Jäncke’s gICA analysis identified a 25–30% reduction in β_1_ (13–20 Hz) power within a component centered on the left superior temporal gyrus (gIC-4) in children with language-specific academic weaknesses; no such deficit was observed in the broader LD-NOS group [41]. This focal hypo-β aligns with fMRI reports of underactivation in the same region during phonological processing tasks and underscores the subtype-specific nature of β abnormalities in LD.

Spectral coherence studies corroborate these observations. Children with LD exhibit elevated frontal and central hypercoherence in the β band, which has been interpreted as excessive, poorly differentiated synchrony [45]. Age-regression analyses further show that the typical developmental pruning of frontal θ-β connections fails to occur in LD, leaving an over-connected, under-specialized network that persists into adolescence [50]. Combined with power data, these findings suggest that β hyperactivity and hypercoherence reflect immature inhibitory control mechanisms within prefrontal circuits.

High-β activity (>20 Hz) is often vulnerable to electromyographic (EMG) contamination; however, the observed anterior β elevations in both Jancke [56] and Lubar et al., (1985) [47] persisted after rigorous artifact rejection procedures, making a purely myogenic origin unlikely. In contrast, the left temporal β reduction was confined to the lower-β range (<20 Hz), a frequency band less susceptible to muscle artifacts [41]. In Eroğlu, (2025) beta/gamma elevations are reported but explicitly flagged as potentially confounded by EMG due to the acquisition system lacking ICA/EMG channels [57].

In sum, the β band reveals a dual abnormality in children with learning disabilities: a frontal excess likely associated with cortical disinhibition and sustained executive effort, and a left temporal deficit that appears linked to phonological-language impairment. Superimposed on the dominant slow-wave phenotype, these β patterns add further specificity to the electrophysiological characterization of LD subtypes and provide insights into the compensatory versus dysfunctional dynamics of the developing brain.

#### 3.6.4. Disrupted Functional Connectivity

Beyond the local power anomalies, children with LD exhibit distinct alterations in large-scale network connectivity, particularly evident when phase synchrony or coherence is examined across developmental stages.

Cross-sectional studies show that younger children with LD display excess delta and theta coherence that spans long distances—bilateral fronto-parietal, fronto-occipital, and left temporo-occipital electrode pairs—producing a markedly “flatter” spatial profile compared to age-matched controls [51]. Among the most severely impaired readers, mean Fisher z-transformed theta coherence at C3–O2 and C4–O1 exceeded control values by approximately 1.2 SD (F = 18–24, *p* < 0.001), while alpha coherence over the same connections was simultaneously reduced by 0.8 SD. This pattern indicates an immature dominance of slow synchrony in circuits that, under typical development, should be transitioning toward faster rhythms [49,51].

This profile persists longitudinally. In a 2.5-year follow-up, typically developing children demonstrated pruning of redundant frontal theta connections and strengthening of posterior alpha coupling. In contrast, children with LD retained—or even intensified—slow-band coherence and failed to establish normative alpha connections, especially between the left temporal and parietal/occipital cortices responsible for reading and orthographic processing [49,50].

Multivariate discriminant models further illuminate this dysconnectivity. Children with LD were characterized by β-band hypercoherence centered on frontal-central hubs, alongside relative hypocoherence in delta and theta bands over the same regions—a dissociation interpreted as inefficient, poorly differentiated executive networks operating in a state of tonic over-drive [45]. The topographic inversion (high-β, low-θ coherence) correctly separated LD from ADHD and controls with >90% accuracy in clinic samples and tracked academic severity in an epidemiological cohort [42,45].

Modern lagged-phase analyses corroborate these findings. In a 19-lead EEG study of 216 children, those with LD exhibited significant reductions in 10.5–12.5 Hz coherence between the left frontal and left parietal cortex, left temporal and right frontal, and right temporal and left occipital regions (all q < 0.05, FDR-corrected) [56]. No group differences remained significant in theta or beta, reinforcing upper-alpha as the most sensitive connectivity marker of academic proficiency—a band associated with long-range, top-down integration in language and memory tasks.

Age-regression analyses provide further developmental context. In typically developing children, posterior alpha coherence (e.g., Cz–P/O pairs) increases steeply with age, while frontal theta coherence declines between the ages of 6 and 16. Children with LD diverged from both patterns: posterior alpha growth was absent, and frontal theta coherence remained stable or increased with age, yielding developmental trajectories that move further away from typical maturation rather than converging on it [50]. These trends suggest a qualitative reorganization—or disorganization—of white-matter pathways and thalamo-cortical loops, rather than a mere delay in timing.

The magnitude of these connectivity anomalies is strongly associated with literacy outcomes. In the Marosi longitudinal dataset, baseline ∆-coherence at C3–O2 explained 46% of the variance in subsequent gains on dictation and reading comprehension tests. Meanwhile reduced upper-alpha coherence in the left temporo-parietal circuit predicted persistent spelling difficulties, even among children who exhibited overall cognitive improvement [49,55].

Taken together, children with LD exhibit a *two-tiered* connectivity profile: (i) over-coupling of slow oscillations that synchronizes widespread regions into an immature, low-frequency rhythm, and (ii) under-coupling of task-relevant alpha networks, particularly those linking left-hemisphere language areas with parietal and occipital sensory hubs. This dual disruption undermines both functional segregation and integration, helping to explain the coexistence of sluggish information processing and excessive compensatory effort that characterizes learning disabilities.

#### 3.6.5. Spatially Specific Source Abnormalities

Source-resolved analyses converge on a remarkably consistent map of “trouble spots” in the brains of children with learning disabilities. Independent component decomposition followed by sLORETA shows that the most prominent slow-wave generator was located on the mesial dorsal frontal wall, extending into the anterior cingulate. In children with broad academic deficits (LD-NOS), the current density in this region was elevated by 35–45% across θ and low-α, whereas children with verbal impairments and neurotypical controls differed only marginally [41].

A complementary FD-VARETA study replicated this frontal excess and, crucially, identified a second, left-dominant focus extending from the inferior through the middle frontal gyrus into the frontal pole and temporal tip. Heightened 3.5–7 Hz output from this peri-Sylvian complex closely tracked phonological underperformance, linking frontal theta excess to language-related difficulties [44].

Posterior regions also contribute but in phenotype-specific ways. A gICA component anchored in the primary and secondary visual cortex (gIC-3) exhibited slowed activity across low-frequency bands only in LD-NOS group—not in children whose difficulties were confined to language—implying that global academic deficits add a visuo-sensory bottleneck atop frontal dysregulation [41]. In contrast, the language-limited subgroup showed a selective 25–30% drop in β_1_ (13–20 Hz) power from a component centered on the left superior temporal gyrus—the region associated with phonological processing—while slow wave activity remained normal or elevated in children with LD-NOS [41]. A further mesial paracentral/superior-parietal node generated extra θ–α power in the broad-impairment group only, consistent with handwriting and arithmetic deficits [41].

Equally striking is what appears to be missing in the LD brain. FD-VARETA revealed that neurotypical controls—but not children with LD—exhibited a robust 10–13 Hz “alpha hotspot” in the right lingual and cuneus gyri. In LD participants, the posterior idling rhythm was replaced by slow-wave activity, flattening the normal occipital dominance [44]. A larger replication that combined source power with lagged phase coherence confirmed the same pattern: overactive slow-wave hubs in mesial frontal and left temporal cortices, underconnected posterior alpha hubs, and a resulting “suboptimal resting-state network” in which long-range integration is impaired [56].

Taken together, these findings portray a circuit-level syndrome: hypersynchronous θ/δ in mesial frontal and left peri-Sylvian regions, focal β underactivation in the phonological temporal area, plus a missing posterior alpha anchor. The relative prominence of each node helps determine whether a child presents with global academic difficulties or a more restricted verbal profile, thus providing a neuroanatomical bridge between heterogeneous classroom symptoms and the electrophysiological signature of learning disability.

#### 3.6.6. Relation to Academic Performance and IQ

A consistent finding across the included studies is that specific EEG abnormalities in children with LD correlate with academic performance and, in some cases, with IQ subscale scores. Several investigations identified high slow-wave power—particularly in the delta and theta bands—as a negative predictor of reading, writing, or general cognitive function. For example, study [42] reported that children with elevated delta/theta power over frontal and central sites tended to score lower on standardized IQ tests. Similarly, study [46] found that increased frontal theta was associated with poorer outcomes, across reading, writing, and arithmetic domains. In a larger sample, study [52] reported strong correlations (r = 0.5–0.75) between composite EEG discriminant scores (incorporating alpha and delta metrics) and academic achievement, especially in reading.

Other studies highlighted more nuanced frequency- and region-specific patterns linked to learning outcomes and cognitive profiles. In study [54], children classified as “poor” or “very poor” in literacy exhibited greater slow-wave activity (delta/theta) and reduced alpha power over anterior, left-hemisphere sites (F3, F7, T3). These EEG differences remained statistically significant even after adjusting for IQ and socioeconomic status, suggesting a direct neurophysiological link to learning outcomes. Likewise, study [46] identified distinct relationships between alpha-1, alpha-2, and specific IQ subscales—higher frontal alpha-2 power correlated with stronger verbal and performance IQ, whereas increased alpha-1 power was inversely related.

Across these findings, children whose EEG profiles deviated most markedly from developmental norms—e.g., with excessive slow activity or diminished alpha in language-relevant areas—tended to exhibit the most pronounced academic challenges, whether in basic reading fluency, comprehension, or more complex tasks such as arithmetic. Moreover, longitudinal studies such as [49,55] suggest that partial normalization of certain EEG measures (e.g., reduction in frontal theta) can accompany academic improvements over time. This supports the idea that EEG markers reflect not only current neurodevelopmental status, but also the evolving neural basis of literacy and learning.

Overall, these results reinforce that resting-state EEG profiles differ consistently between children with LD and their peers, hinting at a potential utility for aiding identification, gauging the severity of learning difficulties, and possibly forecasting academic progress. However, such applications remain speculative until validated by larger, well-controlled studies.

### 3.7. Risk of Bias Assessment

All sixteen studies compared children with LD to typically developing controls using EEG-based measures, generally within cross-sectional or longitudinal observational designs. Overall, we assessed most studies as having a moderate risk of bias, primarily due to potential confounding and selection biases.

Many studies attempted to minimize confounding by excluding children with overt neurological or psychiatric conditions and by matching groups on variable such as age, handedness, or IQ. However, other potentially influential factors—such as socioeconomic status, comorbid ADHD, and parental education—were often reported inconsistently. Few studies employed multivariate statistical adjustments robust enough to account for these variables, leaving open the possibility that unmeasured differences between LD and control groups may have influenced the results.

Selection of participants was another common source of bias. Several studies recruited children with LD from specialized clinics or schools and compared them to convenience samples of typically developing peers. While a few studies used large normative samples, the contrast between clinical and non-clinical recruitment settings may have introduced selection bias, potentially exaggerating observed EEG differences. Moreover, classification of LD status sometimes relied on local definitions, teacher referrals, or performance cutoffs on reading or writing tests. If these criteria were applied inconsistently or lacked external validation, the risk of misclassification bias increased.

As all included studies were observational and lacked assigned interventions, the ROBINS-I domain related to deviations from intended interventions remained minimally applicable. However, differences in services such as tutoring or speech therapy—rarely tracked or standardized—could have influenced outcomes. Missing outcome data posed little risk in the cross-sectional studies, as EEG was typically recorded in a single session. The few longitudinal studies did not report major attrition bias, although limited information was provided regarding differences between children who completed follow-up and those lost to attrition.

Measurement bias was generally low. Quantitative EEG is less prone to subjective influence, and most studies reported using standard electrode montages, artifact rejection protocols, and established spectral analysis procedures. Nonetheless, risk remains if analysts were aware of group status during artifact rejection or if results were selectively reported. Given the broad range of potential EEG features—across multiple frequency bands, coherence measures, and electrode sites—selective outcome reporting cannot be ruled out. No study referenced a prespecified analysis plan or registered protocol outlining specific EEG metrics of interest.

Putting these domains together, the majority of studies were rated as having moderate overall risk of bias under the adapted ROBINS-I framework. A few studies demonstrated stronger methodological features—such as the use of large normative databases, age-adjusted z-scoring, or multi-site recruitment—they still showed gaps in controlling for confounders and potential selection biases. Conversely, several studies were at higher risks due to limited sample sizes, ambiguous LD classification, or insufficient adjustment for underlying medical or demographic variables.

**Figure 2:**
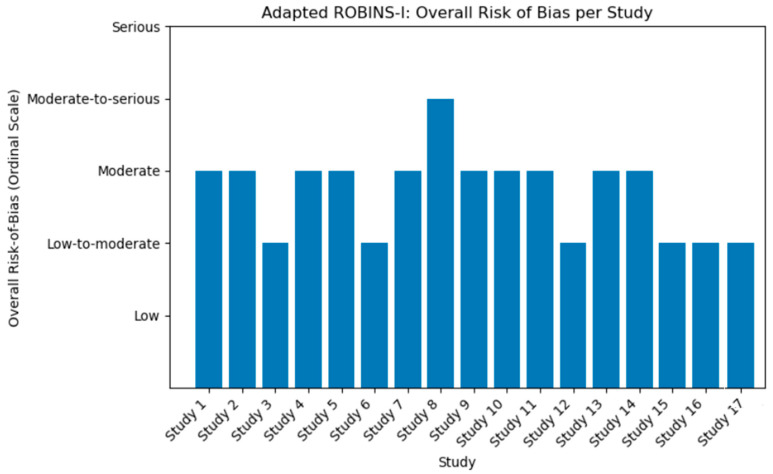
Risk of bias assessment of included studies.

Nonetheless, when considered collectively, the studies present a reasonably consistent picture of EEG abnormalities in children with learning disabilities. These findings should, however, be interpreted with caution regarding causality or precise effect size estimation. Figure 2 summarizes the domain-specific risk of bias ratings based on the ROBINS-I assessment framework.

### 3.8. Metodological Heterogeneity

A critical caveat is the substantial heterogeneity in EEG methodologies across studies. Differences in electrode setups (from 8-channel montages to 19-channel 10–20 caps, with sparse coverage in some early studies), resting-state conditions (most using eyes-closed, but some including eyes-open recordings), and frequency band definitions (inconsistent beta/gamma cut-offs across studies) complicate direct comparison of results. Likewise, some studies focused on power spectra while others analyzed coherence or applied source localization, and artifact rejection protocols ranged from basic manual cleaning to advanced ICA. Finally, the diagnostic definitions of LD varied (DSM-based vs. educational criteria, some excluding comorbid ADHD strictly while others did not), meaning that participant populations were not homogeneous. These disparities likely contribute to variability in findings and highlight the need for standardized EEG recording and analysis pipelines in future research.

## 4. Discussion

Overall, the studies reviewed here converge on the conclusion that resting-state EEG and QEEG measures are robustly associated with learning disabilities (LD) in children, offering valuable insights into the neurophysiological underpinnings of academic underachievement. Across diverse samples, methodologies, and analytic frameworks, several consistent patterns have emerged.

### 4.1. Elevated Slow-Wave Activity as a Marker of Immaturity or Dysfunction

A recurring theme in the reviewed literature is that children with LD exhibit significantly elevated levels of delta and theta power compared to their typically developing peers. This pattern has been consistently reported across multiple studies (e.g., [41,42,43,46,47,53,56]), with slow-wave increases observed across a variety of frequency definitions and electrode sites—most prominently in frontal, central, and parietal regions. In some cases, the effect is robust enough to appear across multiple analytic frameworks, including simple power analyses, ratio-based metrics (e.g., theta/beta, theta/alpha), and neurometric “hit” rates, which reflect how often a child’s resting-state EEG deviates from age-normed expectations.

Several studies interpret this excess in slow-wave power as an index of cortical immaturity or developmental lag. For instance, study [42] demonstrated that children in special education settings—whether classified as learning-disabled or cognitively delayed—consistently exhibited slow-wave power above the levels predicted for their age. Similarly, study [43] showed that when EEG profiles were assessed using developmental regression equations, children with LD or neurological risk factors were significantly more likely to register statistical abnormalities in the delta and theta bands. This finding was echoed in other works: study [47] reported increased theta in 95% of EEG comparisons with control children, and study [56] confirmed a global rise in theta power in children with LD during both eyes-closed and eyes-open conditions.

Mechanistically, these findings align with theoretical models linking elevated slow-wave activity to slower processing and reduced neural efficiency. Because delta/theta naturally decline as part of typical neurodevelopment, persistently high levels of slow-wave power are interpreted as markers of delayed cortical maturation, particularly in networks that subserve higher-order academic skills such as reading or writing. Some longitudinal data suggest that this immaturity may resolve over time. For instance, study [55] found that reductions in delta/theta among children with LD were associated with academic improvement, aligning with typical maturation trajectories. However, a subset of children retained abnormal slow-wave profiles even as they aged, suggesting that in some cases, the observed EEG deviations may reflect not merely delayed maturation, but more enduring cortical dysfunction.

Overall, the finding of elevated slow-wave activity emerges as one of the most robust EEG markers associated with learning disabilities. It transcends methodological differences in electrode montages, frequency-band cutoffs, and signal-processing pipelines. Whether interpreted through traditional spectral analysis, developmental regression models, or independent component methods, the collective evidence supports the view that excess delta and theta—particularly in frontal and central scalp regions—are closely linked to the cognitive and academic challenges characteristic of learning disabilities.

### 4.2. Reduced or Altered Alpha Activity in Language-Related Areas

Many children with LD show atypical alpha activity, particularly over left-hemisphere language regions [41,44,49,54,55]. Across studies, reductions in alpha power (absolute or relative) and altered alpha connectivity are evident at rest and become more pronounced during language tasks.

Study [44] using FD-VARETA reported decreased alpha sources in posterior occipital areas alongside increased slow-wave activity in left-lateralized frontotemporal regions. Study [44] found borderline/abnormal alpha rhythms, especially with brain-risk histories. Elevated frontal and parietal theta/alpha ratios in LD [41] suggest underrepresented alpha relative to slower rhythms, and reduced alpha in left temporal–parietal areas correlates with poorer literacy outcomes [54,55].

These findings fit a maturational-lag account: the EEG of 8–11-year-olds with LD resembles that of younger children, dominated by slower rhythms rather than robust occipital alpha; typically, dominant frequency shifts from theta in early childhood to alpha by adolescence [58]. Less occipital alpha with more frontal theta accords with delayed alpha development, potentially reflecting slower myelination or reduced synaptic density in alpha generators.

Structural factors likely contribute to this. Dyslexia is associated with subtle anomalies in left temporo-parietal regions [59], and during reading, alpha-band abnormalities localize to these sites [60], which often show reduced gray matter or atypical symmetry [61,62]. Thalamic development—central to alpha generation—may also be implicated; in developmental conditions that often co-occur with learning/attention difficulties, lower resting alpha has been linked to reduced thalamic volume [63], supporting a thalamo-cortical contribution.

Top-down regulation of alpha by fronto-parietal attention networks is frequently impaired in LD. Normally, frontal executive regions modulate posterior alpha to gate information flow [64,65]. In LD—often accompanied by attentional deficits [66]—coherence analyses indicate insufficient regulation of long-range alpha coupling and abnormal phase relationships. For example, instead of suppressing frontal–occipital alpha coherence to facilitate visual processing, LD groups may show excessive or erratic connectivity; poor alpha-phase synchronization between temporal and frontal language areas can hinder phonological integration.

Overall, convergent EEG evidence—from spectral power to source localization—indicates altered alpha dynamics in language and attention networks in LD: reduced power, atypical hemispheric balance, and disrupted coherence/phase synchronization, especially over left frontotemporal–parietal regions. These patterns suggest that circuits supporting reading, writing, and broader language skills are underactivated or poorly synchronized in children with LD.

### 4.3. Heterogeneity and Subtypes in LD

A recurring and critical point in the EEG literature on LD is that these children form a heterogeneous group with various cognitive and electrophysiological profiles. While many share common features—such as elevated slow-wave activity or disrupted alpha rhythms—studies repeatedly show that LD is not a one-size-fits-all diagnosis. Instead, it often encompasses multiple subtypes, each with distinct neurophysiological signatures corresponding to specific cognitive deficits.

For instance, study [41] distinguished between children with “LD-NOS” (nonspecific, multifaceted learning difficulties) and those with “LD-Verbal” (language-focused deficits). Although both groups showed elevated theta/beta and theta/alpha ratios compared to controls, more advanced analyses using group Independent Component Analysis (gICA) revealed key differences. Children in the LD-Verbal group showed relatively focal EEG abnormalities in the left temporal cortex—an area associated with language processing—whereas the LD-NOS group showed more diffuse dysfunction across frontal and parietal networks. These patterns suggest that “language-specific” and “generalized” forms of LD may arise from partially distinct cortical mechanisms.

Similarly, study [53] used cluster analysis to cognitive test scores and identified three subgroups within an LD-NOS sample. One subgroup had relatively preserved reading speed and accuracy but poor writing composition; another displayed stronger expressive skills but weaknesses in other areas; a third group showed broad impairments across reading, writing, and arithmetic. EEG analyses confirmed that each subgroup displayed a distinct pattern of slow-wave (delta/theta) elevations and shifts in high-frequency (beta/gamma) activity, reinforcing the idea that specific neurophysiological profiles map onto different cognitive vulnerabilities.

Other investigations [44,52] highlight a parallel point: children with reading disabilities (often classified as dyslexia), those with math-specific difficulties (dyscalculia), and those with more global academic impairments each exhibited unique spectral or connectivity patterns. Even within “pure” dyslexia, multiple EEG markers may vary substantially: some children show left temporal underactivation, while others demonstrate broad frontoparietal dysregulation. These distinctions have meaningful clinical implications, suggesting that interventions should be tailored not just to the broad category of LD, but to each child’s specific neurocognitive and electrophysiological profile.

In sum, the literature consistently underscores that heterogeneity is the rule, not the exception, among children with learning disabilities. The diagnostic label “LD” can obscure diverse developmental trajectories and distinct neurophysiological patterns, ranging from narrowly focused language impairments to diffuse, global delays in neural maturation. Advances in EEG methodology—from source localization to connectivity metrics—are beginning to clarify these subtypes, offering opportunities for more personalized interventions and a deeper understanding of the varied neural mechanisms underlying learning challenges.

### 4.4. Coherence and Connectivity Findings

Beyond spectral power analyses, several studies have investigated the functional connectivity of neural networks in children with LD by examining EEG coherence and related measures (e.g., lagged phase coherence). These connectivity metrics assess the degree to which oscillatory activity across different brain regions is synchronized, providing insights into how efficiently these regions communicate during rest.

A core observation is that children with LD often display less mature or more disorganized connectivity patterns, particularly in the alpha and theta bands. For example, study [49] reported that children with reading and writing difficulties showed abnormal longitudinal trajectories in alpha coherence between frontal, temporal, and parietal sites. While typically developing children exhibited a steady increase in alpha coherence—indicating strengthening interregional connections—those with LD either remained static or showed decreases over time in key language-related connections. Study [50] reinforced these findings, revealing that in controls, coherence levels followed the expected developmental pattern centered on parietal-occipital regions, whereas in LD groups, frontal and interhemispheric connections did not show the expected developmental decline in theta coherence, suggesting an atypical reorganization of cortical networks.

Several works emphasized left-lateralized connectivity deficits. For instance, studies [51,55] showed that children with severe reading or writing impairments consistently exhibited elevated delta/theta coherence and reduced alpha coherence in left temporo-parietal circuits, which are crucial for phonological and orthographic integration. In some cases, these abnormalities persisted into adolescence, suggesting that atypical connectivity patterns may remain stable over time if not remediated.

Study [56] employed lagged phase coherence (LPC) and confirmed that upper-alpha connectivity was particularly sensitive to group differences. Children with LD demonstrated significantly lower LPC in left-lateralized frontal-parietal and temporal-occipital links, signaling impaired long-range communication in networks critical for language, working memory, and attentional control. Notably, these discrepancies were most pronounced in the eyes-closed condition—often considered a baseline connectivity state for resting connectivity—suggesting that the deficits are intrinsic rather than purely task-related.

On a broader level, study [42] and study [43] lend further weight to the notion that connectivity measures, whether coherence or advanced phase metrics, reflect underlying neurodevelopmental processes. In typically developing children, coherence in slow-wave bands (delta/theta) generally diminishes with age as the brain becomes more specialized and efficient, whereas alpha/beta coherence patterns become more pronounced in regions subserving higher-order cognitive functions. In children with LD, these expected developmental shifts are often blunted or even reversed, pointing to disruptions in the maturation of neural networks.

Collectively, these findings suggest that the coherence and connectivity abnormalities seen in LD are not merely a slower version of the normal developmental trajectory but can represent qualitatively different patterns of cortical organization. They also underscore the need to go beyond simple power metrics and examine how different brain areas coordinate their activity. In both clinical and research contexts, functional connectivity measures may help identify distinct subtypes within LD, offering more precise targets for intervention and supporting longitudinal monitoring of brain maturation.

### 4.5. Support for a Maturational Lag Hypothesis—But Tot Exclusively

One of the most longstanding explanations for EEG abnormalities in children with LD is the maturational lag hypothesis, which posits that these children follow a slower-than-typical developmental trajectory rather than displaying a fundamentally different type of brain dysfunction. For instance, study [55] followed children over approximately three years and observed that some with pronounced learning difficulties—who initially showed excesses in delta/theta and deficits in alpha power—shifte toward more normative EEG patterns at follow-up. These improvements aligned with partial academic gains, suggesting that late-maturing cortical networks can, in some cases, “catch up” over time. Similarly, study [49] found that some children with reading and writing difficulties gradually achieved more age-appropriate alpha coherence, although others continued to lag behind, which reinforces the idea that a subset of children with LD may simply be delayed in their developmental trajectory. In study [50], typically developing children showed age-related decreases in frontal coherence decreased with age (indicating greater functional specialization), whereas many in the LD group maintained elevated frontal coherence. Yet repeated measures revealed that a subset of these children with LD did, in fact, begin to show more mature connectivity patterns as they got older.

Still, this evidence must be balanced against a substantial body of work indicating that not all children with LD fit a purely slower version of the normal developmental arc. Rather, many studies point to subgroups of children whose EEG characteristics are qualitatively atypical or remain persistently deviant over time. Studies [41,53] both identified children with broadly impaired neurocognitive profiles who demonstrated stable EEG abnormalities over multiple assessments, suggesting that these patterns were unlikely to normalize with age alone. Likewise, study [43] employed developmental equations to identify children at risk for neurological disorders: while some eventually caught up, a significant fraction did not, retaining clear cortical dysfunction rather than just lagging behind. Study [51] further showed that some children exhibited persistently high slow-wave coherence or persistently low alpha coherence, failing to converge with typical development even into later childhood.

In short, while a maturational delay hypothesis may account for many trajectories within the LD population—particularly for children who eventually narrow the EEG and academic-performance gaps—there exists a subset whose neurophysiological patterns suggest a more fundamental, possibly enduring difference in brain organization. This dual reality highlights the heterogeneity inherent in LD and underscores the importance of individualized assessments and targeted interventions tailored to a child’s specific neurodevelopmental profile.

### 4.6. Diagnostic and Predictive Utility of EEG/QEEG

Several studies in this review suggest that quantitative EEG (QEEG) can serve as a powerful adjunct to conventional clinical evaluations, offering both diagnostic precision and, in some cases, prognostic insight. For instance, study [45] employed discriminant function analysis using nine QEEG features to differentiate children with attention deficit disorders (ADD/ADHD), Specific Developmental Learning Disorders (SDLD), and typically developing controls. In this three-way classification, they achieved 88.7% accuracy for children with ADD/ADHD, 76.1% for controls, and 69% for those with LD—significantly above chance. When directly comparing LD and ADD/ADHD, accuracy climbed above 90%. These findings highlight that even broad EEG metrics, such as frontal theta power or coherence, can be highly discriminative, distinguishing between children with learning-specific issues and those with attentional problems.

In a more focused LD context, study [52] demonstrated the potential of QEEG to both classify LD severity and correlate with academic outcomes. Using a stepwise discriminant model that incorporated power, asymmetry, and coherence variables, they reached over 90% sensitivity and specificity in distinguishing children with LD from controls. Crucially, their EEG-based scores correlated strongly with school performance measures—particularly reading—suggesting that these neurophysiological profiles do more than separate diagnostic groups; they capture meaningful variance in academic skills. The study also introduced an EEG-based severity index, revealing that children with milder academic deficits had intermediate EEG scores between those of controls and severely impaired children, implying a continuum of electrophysiological dysfunction within the LD population.

Other investigations reinforce these findings in varied populations. Study [44], using source localization, reported that focal increases in slow-wave activity predicted poorer language-related performance, thereby providing both a localized diagnostic clue and a rough estimate of language impairment severity. Study [41] offered similar insights for “LD-Verbal” versus “LD-NOS” subtypes; advanced analytic methods such as group Independent Component Analysis (gICA) improved the specificity with which individual learning profiles could be identified. Moreover, some studies hint at predictive value for intervention planning. For example, study [45] found that distinctive QEEG patterns—such as frontal hypercoherence or interhemispheric asymmetries—help predict which stimulant medication would be most effective for children with comorbid attentional symptoms.

Despite these promising results, researchers emphasize the need for careful standardization and robust normative databases. EEG classification accuracy can vary widely depending on factors such as electrode montage, frequency band definitions, artifact rejection protocols, and participant samples. Hence, while QEEG-based classification models have shown high sensitivity and specificity in well-controlled research settings, translating these tools into routine clinical practice demands consistent methodologies, larger cross-cultural validation samples, and clear guidelines for interpreting results. QEEG shows potential to differentiate LD from typical development, but diagnostic precision remains to be conclusively demonstrated.

### 4.7. Left-Lateralized Abnormalities and Language Networks

A central theme in the reviewed studies is that children with LD—particularly those whose deficits center on reading, writing, or other language-related skills—often show more pronounced EEG abnormalities in the left hemisphere. This lateralization aligns with the left hemisphere’s well-established role in phonological decoding, semantic processing, and orthographic integration.

Multiple studies provide converging evidence. Study [41] found that children with language-based learning problems (“LD-Verbal”) showed focal EEG disturbances localized to the left temporal cortex, as revealed through gICA and source localization techniques. While children with broader LD (“LD-NOS”) displayed more diffuse abnormalities, the left temporal region was a consistent site of dysfunction in language-impaired subgroups. In a similar vein, study [44], using FD-VARETA, discovered that theta-band overactivation was most pronounced in the left inferior and middle frontal gyri, extending into the anterior cingulate and left temporal pole—regions intimately involved in language production and phonological awareness.

Further evidence comes from study [53], where the most severely impaired subgroup—showing widespread deficits across reading, writing, and arithmetic—exhibited elevated slow-wave activity across left frontotemporal and parietal regions. These increases correlated with lower performance in language tasks, suggesting that cortical regions supporting phonological and semantic processing may be underactive or developmentally delayed in this subgroup. Moreover, study [54] reported that poor or very poor readers had increased slow-wave power and reduced alpha power specifically over left frontal-temporal electrodes (F3, F7, T3), while study [55] demonstrated that alpha and beta patterns in left frontotemporal regions were particularly predictive of long-term reading and writing outcomes.

This emphasis on the left hemisphere also emerges in connectivity analyses. Studies [49,50,51] frequently point to attenuated alpha coherence or elevated slow-band coherence in left temporo-parietal circuits—areas critical for phonological decoding and orthographic processing. Study [56] similarly observed that upper-alpha connectivity deficits in left-lateralized frontal, parietal, and temporal networks contributed to impaired integration of language-related functions.

Collectively, these findings reinforce the conclusion that learning disabilities—especially those affecting language—are frequently associated with selective disturbances in the left hemisphere’s cortical organization and interregional connectivity. While not every child with LD displays clearly left-lateralized abnormalities, this pattern appears frequently enough to be considered a hallmark of language-specific challenges. This observation aligns with broader neuroimaging evidence showing structural and functional anomalies in left temporo-parietal pathways among children with reading and writing difficulties. As such, the focus on left-hemisphere dysfunction not only helps explain common phonological and orthographic impairments in LD populations but also offers a promising avenue for developing more targeted diagnostic and intervention strategies.

### 4.8. Neuroinflammation Signature Based on EEG

Eroğlu explicitly interprets the theta/alpha/gamma profile as a neuroinflammation-linked signature and reports very high ANN-based separability; while intriguing, this mechanistic attribution remains hypothesis-level and requires multimodal biomarkers (e.g., cytokines) and longitudinal designs for confirmation [57].

## 5. Possible Interventions in LD Based on EEG Results

When consistent patterns of altered EEG activity are identified in a given disorder, specialized interventions can be developed to target this dysregulation [67]. One such method is EEG neurofeedback training, which involves the conscious modulation of EEG activity using conditioned learning through receiving visual and auditory gratifications in real time [68,69]. In EEG neurofeedback training, participants wear EEG sensors that record brain activity at several scalp locations [70]. Based on the observed deviations, the therapist sets a therapeutic goal—either to strengthen or inhibit specific brainwave patterns [70]. When the participant’s EEG activity shifts changes in the desired direction, they receive immediate feedback, such as visual or auditory stimuli (e.g., elements in a video game) [71]. Over time, this reward-based training facilitates the learning of new neural patterns, which can lead to long-term changes in EEG activity [72].

Several studies have explored EEG neurofeedback training to treat learning disorders. Protocols typically aim to reduce signs of cortical underarousal and enhance neural efficiency. As discussed, many children with LD show globally elevated theta power or theta/alpha ratios in resting-state EEG. Therefore NFB often targets the suppression of slow-wave activity at the scalp location(s) where these abnormalities are most pronounced [73,74]. Studies employing this approach have documented clear neurophysiological improvements. For example, Fernandez et al. [73] applied 20 sessions of theta/alpha down-training in children with high theta/alpha ratios and found broad-band EEG power reductions (delta, theta, alpha, and beta) in the NFB group post-treatment, changes reflecting a generalized normalization of brain activity not observed in placebo controls. Likewise, a sham-controlled trial by Martínez-Briones et al. [75], involving 30 NFB sessions, reported significant task-related EEG changes: children who received NFB showed decreased frontal theta power and increased beta and gamma activity during a working memory task—indicating more efficient cortical engagement.

Source-localization analyses provide further evidence that NFB can induce region-specific alterations in neural oscillations. In an exploratory study using EEG current source density, children with LD who received NFB showed delayed but pronounced reductions in cortical theta sources, particularly in frontal and cingulate regions. These changes were accompanied by strengthened alpha and beta activity in frontal and temporal areas two months after training [74]. These neurophysiological findings substantiate that NFB can remediate the sluggish EEG profiles observed in children with LD, effectively tuning brain networks for more efficient information processing.

Therapeutically, modifying the EEG through NFB yields measurable gains across multiple cognitive and behavioral domains in children with LD. Controlled trials have demonstrated improvements in core intellectual and executive functions following NFB. In the study by Fernandez et al. [73], children who received NFB showed significantly greater improvements on the Wechsler Intelligence Scale for Children (WISC) scores relative to placebo controls, suggesting enhanced overall cognitive function. Similarly, Linden et al. [76] reported a mean increase of ~9 points in IQ (Kaufman Brief IQ) after 40 sessions of beta-enhancement/theta-suppression training in a sample with LD/ADHD, alongside reductions in parent-reported inattentive behaviors.

Benefits extend to academic performance as well. In one study, children in the NFB group showed significantly higher post-training scores in reading and mathematics compared to controls [77]. These academic gains were accompanied by enhanced attention and working memory—for instance, faster response times on the Sternberg memory test indicated improved working memory efficiency [75]. Early case studies also documented notable improvements in learning-related abilities following EEG biofeedback. Tansey [78], for example, observed substantial increases in WISC IQ scores (Full-Scale, Verbal, and Performance) and a normalization of the Verbal–Performance IQ discrepancy in children with LD undergoing a sensorimotor rhythm (≈14 Hz) up-training protocol.

Such converging evidence from diverse studies attests that NFB-induced EEG normalization translates into better cognitive performance, attentional control, and academic output in LD populations.

Beyond cognitive metrics, NFB may also ameliorate behavioral and psychosocial difficulties commonly associated with learning disorders. Children with LD frequently struggle with low self-esteem and anxiety related to academic failures, and encouragingly, NFB appears to confer improvements in these areas. In an exploratory study, Martínez-Briones et al. [77] found that NFB not only enhanced academic performance but also significantly improved global self-concept in 8–11 year-old children with LD. Treated children reported greater self-worth across domains such as physical appearance, popularity, and happiness, and also showed reductions in anxiety. In contrast, controls and wait-list groups experienced no comparable benefits [77]. These findings suggest that by improving children’s cognitive functioning and school competence, NFB can produce secondary gains in emotional well-being and confidence.

Behavioral regulation may also improve following NFB. In addition to gains in attention seen among children with co-occurring ADHD symptoms [76], parents and teachers often report better focus and reduced impulsivity after NFB training—qualitative observations that align with objective attentional improvement. Importantly, no serious adverse effects have been reported in pediatric studies to date. Some placebo-controlled designs noted modest improvements in sham-training groups due to non-specific factors, underscoring the importance of adequate control conditions when evaluating behavioral outcomes [77]. Overall, the therapeutic effects of NFB in LD extend beyond test scores, encompassing meaningful improvements in daily functioning and self-perception.

A growing body of evidence supports NFB as a promising intervention for learning disorders, with both theoretical significance and practical implications. The efficacy of NFB in addressing EEG abnormalities reinforces the hypothesis that some LDs reflect neurodevelopmental delays—or “brain dysmaturity”—that may be partially remediated through targeted brain training [75,77]. By directly conditioning brain activity toward more normative patterns, NFB offers a novel strategy to induce neuroplastic changes that may not be achievable through traditional educational interventions alone. This creates opportunities for integrating NFB into multidisciplinary approaches to LD, where it could complement academic remediation by enhancing neurocognitive foundations for learning.

At the same time, research emphasizes the need for careful optimization of NFB protocols to ensure maximum benefit for children with LD. For instance, the modality of feedback delivery may influence outcomes. One study found that auditory feedback (tones presented with eyes-closed) was more effective than visual screen-based feedback for improving cognitive performance, although both modalities led to EEG changes [78]. This suggests that minimizing distractions and using simplified reinforcement signals may enhance self-regulation learning [78,79]. Individual differences in neurophysiology also matter. Recent findings suggest that the individual alpha peak frequency may predict NFB responsiveness: children with more age-typical alpha rhythms tended to show greater EEG normalization after training than those with atypically low alpha frequency [80]. These results highlight the need for personalized NFB protocols, where treatment parameters (e.g., reward frequency bands or threshold criteria) are tailored to each child’s baseline EEG profile.

Finally, longitudinal observations indicate that the benefits of NFB can be durable. Follow-up assessments conducted more than two years after treatment have noted that cognitive and EEG improvements are largely maintained, supporting the view that NFB induces lasting neural reorganization rather than transient placebo effects [74,79].

The findings summarized in this review provide a solid foundation for designing EEG-informed neurofeedback interventions aimed at reducing abnormal slow-wave activity (theta/delta) and reinforcing the faster rhythms (alpha and low-beta) linked to more mature cortical processing in children with LD. Protocols can be configured to address the specific EEG anomalies characteristic of different LD subtypes. For example, many children with LD show a heightened theta/beta ratio (TBR) in frontal regions, reflecting pronounced slow-wave activity that may impair attention. A commonly used intervention targets midline-frontal or frontocentral sites, rewarding each instance of a drop in theta amplitude and a concurrent rise in beta amplitude. By reducing TBR, such training seeks to improve attentional control and cognitive efficiency critical for managing classroom demands.

Another well-supported strategy is to focus on the theta/alpha ratio (TAR), especially in children with reading or spelling difficulties who show high theta and low alpha in frontal-temporal or parietal areas. Here, by rewarding lower theta or higher alpha amplitudes over these leads, therapists can encourage a transition toward more age-appropriate cortical rhythms. For children with LD exhibit coherence deficits in the alpha band—particularly in frontotemporal or temporo-parietal connections vital for language and reading—two-channel coherence training may help strengthen network synchronization between critical for reading and language processing.

In cases with more generalized EEG anomalies, a multiphase protocol may be effective. This can begin with dampening global slow-wave activity (especially frontal or central theta/delta) and then shift toward enhancing alpha or low-beta rhythms in posterior regions to bolster attention and sensory integration. Practitioners may also consider eyes-open training protocols that monitor parietal and occipital EEG patterns during reading or writing tasks, thereby mimicking classroom conditions. Across all these approaches, source-localization or advanced analysis methods (e.g., FD-VARETA) can guide precise electrode placement by identifying the most aberrant cortical regions in each child. By tailoring NFB protocols in this way, practitioners can more precisely target the neurophysiological underpinnings of LD, potentially achieving more durable improvements in literacy, attention, and overall academic performance.

## 6. Limitations and Future Directions

Despite converging evidence for a robust electrophysiological phenotype in children with LD, the existing body of research is constrained by several methodological and conceptual shortcomings that limit generalizability and translational impact.

### 6.1. Sample-Related Constraints

The evidence base remains dominated by small, convenience cohorts recruited from single clinics or special education classrooms. Half of the papers included fewer than fifty participants with LD, and only two reported a priori power calculations, leaving most analyses statistically underpowered and prone to unstable effect size estimates. The sex distribution is also notably skewed: Lubar’s task study, for example, investigated 103 boys but no girls [47], and Thatcher’s discriminant analysis contained nearly 80% male participants [52]. Such imbalances obscure potential sex-specific developmental trajectories, which have begun to emerge in larger normative EEG datasets [50,51].

Diagnostic criteria also vary widely, ranging from placement in a “special-needs” classroom [42] to DSM-III-defined Specific Developmental Learning Disorder [41] or locally defined “very poor readers” [54]—Exclusion criteria for ADHD, language disorder, or borderline IQ are inconsistently applied. Consequently, many samples amalgamate children with qualitatively different neurocognitive profiles, inflating within-group variability and reducing comparability across studies.

Cultural and socioeconomic representation is equally narrow. With the exception of Ahn’s cross-cultural neurometric validation in Barbados [43], all studies were conducted in North- or Latin-American urban settings. Only two explicitly measured socioeconomic status [54], and none adjusted EEG outcomes for environmental disadvantage. These limitations collectively hamper generalizability, impede meta-analytic synthesis, and increase the risk that putative biomarkers reflect local referral biases or contextual deprivation rather than true neurobiological features of LD.

To address these issues, future investigations should prioritize multi-site recruitment, ensure sex-balanced samples, and adopt harmonized DSM-5/ICD-11 diagnostics. Moreover, participant stratification by socioeconomic background will be essential to disentangle genuine pathophysiological signals from demographic noise.

### 6.2. Technological and Analytic Limitations

Most of the reviewed investigations relied on instrumentation and signal processing pipelines that would now be judged rudimentary. Ten of the sixteen studies employed the classic 10–20 montage with 19 or fewer scalp electrodes; several early studies analyzed as few as eight channels, fixed to midline and homotopic sites [42,43]. Such sparse spatial sampling blurs cortical sources, amplifies volume-conduction artifacts, and precludes reliable mapping of the temporo-parietal hubs that become visible in high-density arrays. Only two studies combined high-density recordings with source reconstruction methods (sLORETA or FD-VARETA) [41,44], illustrating how rarely advanced inverse modeling is applied in pediatric LD research.

Signal bandwidth and frequency band definitions are also inconsistent. For example, “beta” is variously set to 13–22 Hz [52], 17.5–25 Hz [42], or 13–30 Hz [56]; gamma activity is ignored in all but one study [53], and infraslow rhythms (<1 Hz) are universally filtered out at acquisition. Such discrepancies hinder cross-study comparability and may obscure frequency-specific markers, such as theta–gamma coupling, which more recent research links to phonological processing.

Artifact management remains a weak point. Early studies relied almost exclusively on visual epoch rejection, leaving high-beta signals susceptible to muscle contamination and delta to residual eye movements; only the 2019 dataset applied systematic independent-component analysis to strip ocular and myogenic noise [56].

Connectivity metrics are similarly dated. Most studies continue to use classic magnitude-squared coherence, which is sensitive to reference electrode effects and instantaneous field spread. More sophisticated metrics—such as lagged-phase coherence, imaginary coherence, and graph-theoretical approaches—are rarely used, despite their ability to better isolate genuine neuronal interactions. Only one sLORETA-based study employed network-level statistics, and no study examined dynamic functional connectivity or implemented machine learning classifiers that integrate power and phase information.

To overcome these limitations, future research should adopt ≥64-channel EEG caps, publish raw and pre-processed datasets in open repositories, standardize filter settings and band nomenclature, deploy automated ICA plus surface Laplacian transforms, and shift from scalp coherence to source-level lagged or directionality-sensitive metrics. Such technological upgrades will sharpen localization, reduce artifact contamination, and ultimately allow laboratories worldwide to replicate—and meaningfully compare—oscillatory fingerprints of learning disability.

### 6.3. Design Shortcomings

The design of nearly all reviewed studies is inherently static: fourteen of the sixteen datasets captured only a single, eyes-closed snapshot of brain activity, offering no insight into how oscillatory patterns evolve as children develop or respond to intervention. Only two studies tracked participants longitudinally, and both followed modest cohorts (<50 children) across just one additional time point three years later, leaving the most critical developmental inflection—early adolescence—largely unexplored. Task conditions are similarly under-represented: Lubar’s 1985 experiment remains the only one to examine LD-related electrophysiology during active tasks such as reading, calculating, or problem-solving. As a result, it remains unclear whether resting-state EEG phenotypes predict online cognitive load, error monitoring, or responsiveness to remediation.

Another major limitation is the near-complete absence of multimodal convergence. None of the EEG cohorts were scanned with MRI to validate source generators against cortical thickness, myelination, or white-matter connectivity; nor were genetic or physiological data (e.g., saliva or blood samples) collected to link oscillatory delays with genetic risk alleles or stress biomarkers. Likewise, no study incorporated EEG endpoints into controlled treatment trials—be it structured literacy programs, neurofeedback, or non-invasive brain stimulation—leaving open the question of whether the identified rhythms are modifiable or behaviorally relevant. Finally, ecological validity is low: recordings typically occur in shielded laboratories, whereas learning difficulties arise in noisy, dynamic classrooms, where attention, posture, and motivation fluctuate widely.

To address these design limitations, future studies should adopt accelerated longitudinal designs that sample children annually from preschool to late adolescence. EEG assessments should be complemented with task-based recordings, eye-tracking, fine-grained behavioral measures, and classroom observation. High-density EEG should be fused with structural and diffusion-weighted MRI to anchor oscillatory hubs in anatomy. Finally, EEG markers should be embedded as primary or secondary outcomes in randomized trials of educational or neuromodulatory interventions. Only such developmentally sensitive, multimodal protocols will reveal whether current electrophysiological fingerprints are causes, correlates, or consequences of learning disability—and whether they can guide genuinely personalized intervention.

### 6.4. Statistical and Translational Gaps

Although several groups report striking effect sizes and classification accuracies, statistics underpinnings are often fragile. Discriminant analyses claiming 95–99% sensitivity and specificity typically mine large numbers of EEG variables in samples too small to support such parameter density. These models often rely on liberal stepwise selection and validate performance only by split-half or leave-one-out methods on the same dataset. Without nested cross-validation—or, better, external replication cohorts—such results likely reflect overfitting. Indeed, the few studies that attempted genuine hold-out replication saw accuracy decline by 15–25%.

Other statistical weaknesses persist. Power calculations are rarely reported, multiple-comparison corrections are inconsistently applied, and null findings—such as the absence of delta or gamma effects—are seldom disclosed, contributing to publication bias. The field thus lacks a clear sense of which EEG features are robust, replicable, and meaningfully linked to LD phenotypes.

Clinical specificity remains equally uncertain. Only one study compared LD directly with ADHD and showed that slow-wave excess is less diagnostically distinct than previously believed. Comparisons with other neurodevelopmental conditions—such as autism spectrum disorder, specific language impairment, developmental coordination disorder, or anxiety—are entirely absent. Consequently, it is unclear whether the widely cited markers such as elevated θ/α or θ/β ratios are specific to LD or reflect a broader, trans-diagnostic maturational delay. No study has tested whether EEG-informed screening enhances educational planning, predicts individual education plan outcomes, or improves cost–effectiveness compared to conventional psychometrics. Although EEG predictors of stimulant response in ADHD offer some translational promise, equivalent trials for literacy interventions, neurofeedback, or transcranial alternating current stimulation tACS have yet to materialize. So, it is important to note that many rs-EEG abnormalities in LD (e.g., elevated theta/beta ratios or slow-wave excess) are not specific to learning disorders—similar patterns are reported in ADHD and other developmental conditions. This raises the possibility that these markers reflect a broader neurodevelopmental immaturity rather than a unique signature of LD per se.

To bridge these gaps, future research must adopt preregistered analysis plans, use nested or multi-site cross-validation, and report full confusion matrices and confidence intervals for effect sizes. Datasets should be shared openly, including both code and de-identified participant data. Studies should include multiple neurodevelopmental multiple comparison groups across neurodevelopmental conditions to isolate disorder-specific markers from broader maturational indicators. Crucially, prospective intervention trials should test whether baseline EEG features can stratify children into subgroups differentially responsive to validated reading programs or neuromodulation therapies. Only by addressing these statistical and translational challenges can pediatric EEG move beyond descriptive research to inform actionable, precision education.

### 6.5. Open Questions Worth Pursuing

Several conceptual avenues remain largely unexplored and warrant systematic investigation. First, although most research has focused on band-limited power, reading and language proficiency depend on dynamic cross-frequency interactions. Determining whether aberrant theta–gamma or beta–gamma phase-amplitude coupling serves as a more precise index of phonological parsing than conventional slow/fast ratios should be a research priority. Second, given that most cohorts are male-dominated and prepubertal, little is known about how pubertal hormones shape key oscillatory changes—such as frontal beta surge or the posterior alpha decline. Mapping sex-specific developmental trajectories could help explain differences in the onset, persistence, and resolution of reading disabilities between girls and boys. Third, oscillatory maturation is unlikely to be purely experiential; integrating EEG with genetic and environmental data could clarify how literacy-risk alleles such as DCDC2 or KIAA0319 interact with language exposure, sleep quality, or socioeconomic stress to accelerate or impede cortical pruning. Fourth, current “learning disability” diagnoses conflate children with diverse brain profiles; multimodal clustering that combines dense-array EEG, MRI connectomics, and behavior could yield biologically coherent subtypes, each with distinct prognoses and optimal treatment pathways. Finally, translation will depend on developing biomarkers that function outside laboratory settings. Testing whether low-density, wearable dry-EEG devices can reliably capture the slow/fast ratios and alpha topography in noisy classrooms could enable large-scale screening and real-time monitoring of remedial progress. Pursuing these questions will help move pediatric EEG from description toward a mechanistic and practically useful science of learning.

### 6.6. Practical Implementation Challenges

Even if robust EEG biomarkers for LD are identified, several hurdles must be overcome before they can be deployed in schools or clinics. Cost is a major factor—high-density EEG systems and setup can be expensive and may not be feasible in resource-limited educational settings. Standardization is also critical: results will only be interpretable if recording protocols (e.g., electrode placement, eyes-open vs. closed conditions, artifact handling) are uniform across sites. Additionally, administering and interpreting EEG in young children requires specialized EEG technician training and child-friendly procedures to ensure data quality. There are also logistical challenges to integrating EEG into school systems or pediatric offices—for instance, finding time and space for recordings amid regular school activities, and addressing potential stigma or parental consent issues. These implementation barriers mean that, even if the science of rs-EEG biomarkers progresses, translating it into routine early screening for LD will require concerted efforts in training, infrastructure, and protocol development.

## 7. Conclusions

Resting-state electroencephalography offers a coherent, neurally grounded view of the biological underpinnings of learning disabilities in childhood. Although the 17 primary studies reviewed here vary in sampling frames, diagnostic definitions, and analytic methods, they converge on a remarkably consistent electrophysiological pattern across diverse cohorts and methods. Children with LD show a pervasive excess of slow oscillations—delta and theta—over mesial-frontal, fronto-central, and left peri-Sylvian cortices, leading to elevated theta/alpha and theta/beta ratios that correspond to an electrodevelopmental delay of approximately two to four years. This slow-wave dominance is accompanied by attenuation or anterior displacement of the posterior alpha rhythm, particularly in left temporo-parietal regions involved in phonological and orthographic processing. Focal beta-band abnormalities also emerge, distinguishing compensatory frontal activation from language-specific underactivation in temporal areas. Connectivity analyses reveal a dual disturbance: on the one hand, the immature over-coupling of slow waves locks widespread regions into low-frequency synchrony; on the other, the under-coupling of upper-alpha networks hampers efficient communication between left-hemisphere language hubs and posterior sensory areas. Source-resolved analyses anchor these oscillatory signatures to discrete cortical generators, reinforcing the association between heterogeneous behavioral symptoms and specific circuit-level dysfunctions.

Taken together, these findings support a hybrid explanatory model. For many children, the EEG profile reflects a maturational lag that converges with typical development over time; however, a substantial minority exhibits qualitatively atypical network organization that is unlikely to resolve without targeted intervention. Resting-state EEG emerges as a robust group-level indicator of neurophysiological differences in children with learning disabilities. It shows promise as a stratification tool that might help identify clinically relevant subtypes and potentially predict academic outcomes, but current evidence is derived from small, heterogeneous studies with moderate risk of bias. These EEG markers are not yet ready for routine diagnostic or prognostic use. Realizing their clinical potential will require large, multi-center studies with standardized methods and demonstration in trials that EEG-guided approaches improve outcomes.

Before such promise can be realized in clinical and educational contexts, several key limitations must be addressed. Current knowledge is based largely on small, clinic-based samples with sex imbalances, inconsistent exclusion criteria, sparse electrode coverage, and outdated connectivity metrics. Future research must adopt multi-center, longitudinal designs that apply harmonized DSM-5/ICD-11 definitions, employ dense-array recordings aligned with MRI, genetic, and environmental measures, and follow preregistered analytic pipelines with rigorous cross-validation. Equally important will be randomized trials that embed EEG markers within evidence-based literacy, numeracy, or neuromodulatory interventions—trials capable of demonstrating whether EEG phenotypes can guide treatment selection and monitor brain-level change.

If these methodological and translational challenges are met, resting-state EEG could evolve from a descriptive neuroscience tool into a scalable biomarker platform—one that flags neurodevelopmental risk before academic failure becomes entrenched, supports tailored intervention, and objectively monitors progress across the lifespan. At present, however, these applications remain aspirational. Researchers and clinicians must resolve the aforementioned limitations and prove that EEG can reliably guide interventions in order to realize this vision. Such advancements would represent a major step toward ensuring that learning disabilities are manageable differences rather than determinants of lifelong educational and occupational disadvantages.

## Figures and Tables

**Table 1:** Studies included in the review.

Study	Sample/Condition	N	Control/Comparison	EEG Setup	Analysis	Key EEG Findings
[41]	LD-NOS, LD-Verbal, HC (age ≈ 9 y)	25/29/32	Within-group & HC	19-ch; EO + EC	TBR & TAR; gICA + sLORETA	↑TBR/TAR in both LD groups; LD-NOS shows widespread δ/θ/α excess across 5 gICs; LD-Verbal shows focal β1 ↓ in left temporal (language)
[42]	German school sample: MR, LD, Norm	47/26/158	Norms & MR	8-ch; EC	Log power z-scores; PCA	LD: moderate frontal δ/θ ↑ (≈+0.5 SDS); α2/β2 occipital ↓; supports delayed maturation profile
[43]	Multi-site neurometric (Groups 1–5)	310 Norm, 163 LD-avg-IQ, 143 LD-borderline IQ	Age-norm eqns	8-ch; EC	Age-regressed Z; “hits” > 1.64 SD	>50% of LD kids flagged abnormal; parieto-occipital δ/θ ↑ & α ↓; heterogeneous EEG phenotypes
[44]	LD-NOS vs. HC (7–11 y)	46/25	HC	19-ch; EC	FD-VARETA source	LD: frontal θ excess (left > right); HC: occipital α dominance; clear frontal-temporal θ generators in LD
[45]	ADD/ADHD vs. LD vs. Norm	407/242/310	Three-way	19-ch; EC	QEEG discriminant	Model classifies ADD 88%, LD 69%, Norm 76%; LD pattern = frontal/central δ ↑ + incoherence
[46]	Brazilian LD clinic vs. HC	36/36	Matched HC	19-ch; EC	FFT (AP & RP); Bonferroni	LD: absolute δ/θ/α1 ↑ widespread; RP: θ ↑ & α2 ↓ fronto-central; α2 RP ↔ IQ (+), α1 RP (–)
[47]	LD (no ADHD) vs. Norm during tasks	69/34	Baseline & tasks	6 leads; EO baseline	4-way ANOVA; FFT	LD show 4–8 Hz & 6–10 Hz power ↑ in 95% of conditions; discriminant 98% accurate with spectral vars
[48]	LD vs. good schoolers (visual EEG)	61/61	Good performers	Clinical EEG	Visual grading	Abnormal EEG (high-amp α, focal paroxysms) far more common in LD; eye-closure paroxysms only in risk-history LD
[49]	Longitudinal coherence (good/mild/severe)	18/17/11	Two recordings 2.5 y apart	15-ch; EC	Coherence ANOVA	Good readers: α coherence ↑ with age; severe LD: θ coherence ↑ & α coherence ↓ ⇒ divergent maturation
[50]	98 HC vs. 54 LD (6–16 y)	98/54	Age-sex matched	15-ch; EC	Linear regressions of coherence vs. age	HC: posterior Cz-centered coherence ↑; LD: no Cz maturation, ↑ frontal α/β coherence; atypical trajectory
[51]	Younger & older cohorts (ped1–3)	84 total	Group × age	15-ch; EC	Coherence ANOVA; DFA	Younger: δ/θ coherence ↑ in severe LD; α coherence ↓; model classifies severe LD 100%. Older: differences smaller but still discriminant
[52]	Large qEEG LD vs. Norm	58 LD (train + rep)/277 Norm	Split-half validation	19-ch; EC	10-var discriminant	Sens 94–97%, Spec 99%; EEG severity index tracks WRAT & WISC scores linearly
[53]	Mexican LDNOS subgroups (G1–G3)	85	Within LD	19-ch; EC	k-means clusters; narrow-band AP	G3 (worst): δ/θ ↑ left FT/PO, γ ↓; G1/G2 show higher α/β or γ consistent with better literacy
[54]	EEG vs. literacy (good→very poor)	81	4 perf. levels	14-ch; EC	Age-Z spectral; Canonical corr.	Poor readers: θ ↑ & α ↓ over left F/T; EEG predicts literacy (up to 78% correct) independent of IQ/SES
[55]	3-year follow-up good/mild/severe	49 total	Baseline vs. follow-up	15-ch; EC	Repeated-measures FFT	All groups: δ/θ ↓ & α RP ↑ with age; severe LD retain posterior θ RP ↑; supports maturational-lag
[56]	LD vs. HC (spectral + LPC)	84/132	HC	EO & EC, 19-ch; ICA clean	FFT AP; sLORETA LPC	LD: global θ AP ↑ (d > 0.8); frontal β ↑ EO; EC upper-α LPC ↓ in F–P & T–O networks ⇒ impaired integration
[57]	LD vs. HC	100/100	HC	EO, 14 ch	Artificial neural networks	Reduced posterior/overall alpha as part of a putative neuroinflammatory EEG signature

Abbreviations: LD—learning disability; LD-NOS—learning disability not otherwise specified; LD-Verbal—LD with verbal deficits; HC—healthy control; ID—intellectually disabled; TBR—theta/beta ratio; TAR—theta/alpha ratio; gICA—group independent component analysis; sLORETA—standardized low-resolution electromagnetic tomography; FD-VARETA—frequency-domain variable resolution electromagnetic tomography; QEEG—quantitative EEG; AP—absolute power; RP—relative power; SDS—standard deviation score; PCA—principal component analysis; LPC—lagged phase coherence; EO—eyes open; EC—eyes closed; FFT—fast Fourier transform; ANOVA—analysis of variance; DFA—discriminant function analysis; WRAT—wide range achievement test; WISC—Wechsler Intelligence Scale for Children; SPT—School Performance Test; ENI—Infant Neuropsychological Evaluation; ADD—attention deficit disorder; ADHD—attention deficit/hyperactivity disorder; SLD—specific learning disorder; SDLD—specific developmental learning disorder; IQ—intelligence quotient; δ—delta band; θ—theta band; α—alpha band; β—beta band; γ—gamma band.

## Data Availability

No new data were created or analyzed in this study. Data sharing is not applicable to this article.
